# Tobacco Industry Efforts to Respond to Smoke-Free Policies in Multi-Unit Housing: An Evaluation of Tobacco Industry Documents

**DOI:** 10.3390/ijerph19053053

**Published:** 2022-03-05

**Authors:** Joshua Miller, Maya Vijayaraghavan

**Affiliations:** 1 Cardiovascular Research Institute, School of Medicine San Francisco, University of California, San Francisco, CA 94143, USA; 2 Division of General Internal Medicine, Zuckerberg San Francisco General Hospital, University of California, San Francisco, CA 94143, USA; maya.vijayaraghavan@ucsf.edu

**Keywords:** tobacco control, multi-unit housing, secondhand smoke

## Abstract

The tobacco industry’s efforts to undermine clean indoor air policies in the hospitality industry, public spaces and workspaces is well documented, but less is known about their efforts to respond to the implementation of smoke-free policies in multi-unit housing (MUH). From 1988 to 2018, public and private multi-unit housing properties voluntarily implemented smoke-free polices in their buildings. We searched the UCSF’s Truth Tobacco Industry Documents Library to examine whether the tobacco industry responded to the implementation of these smoke-free policies in MUH using the same strategies they deployed to respond to smoke-free policies in other industries. We found that the tobacco industry used two primary strategies to respond to smoke-free policies in multi-unit housing: (1) distortion, which included funding studies that downplayed the link between SHS and asthma among low-income, inner-city MUH residents; and (2) deflection, which included engaging in corporate responsibility for youth living in low-income MUH. Despite these efforts, local jurisdictions continued to voluntarily implement smoke-free policies in MUH, pointing to a potential counter strategy to the tobacco industry influence.

## 1. Introduction

The tobacco industry’s efforts to challenge indoor smoke-free policies are well documented [1,2], but less is known about their efforts to respond to smoke-free policies in multi-unit housing (MUH). Understanding the tobacco industries’ attempts to respond to smoke-free policies in MUH is important because one-third of all renters, including those living in MUH, are exposed to secondhand smoke (SHS) [3]. SHS, which causes 41,000 deaths each year in the United States [4], can travel from smoking units to non-smoking units along air ducts, through the walls, floors, cracks, elevator shafts, plumbing and even electrical routes [5]. Without smoke-free policies, non-smoking MUH residents are at risk for harms related to SHS exposure.

Smoke-free policies in MUH is particularly relevant to addressing tobacco-related health equity [5]. Over 35% of public MUH residents are disabled, 41% are children, and 32% are elderly, all populations that are vulnerable to SHS exposure and experience heightened morbidity and mortality from SHS [1]. Moreover, low-income MUH residents have a higher prevalence of tobacco use than the general population, due in part to tobacco industry targeting of these groups [6]. Therefore, understanding how the tobacco industry responded to the implementation of smoke-free policies in MUH can be critical to reducing disparities in SHS exposure among low-income and marginalized populations in the U.S.

Despite 26% (79.2 million) of the U.S. population living in MUH [7], and facing heightened risk of exposure to SHS, most cities and states do not require smoke-free policies in MUH [8]. In a national study of MUH residents in 2010, only 29% reported living in smoke-free buildings, and among residents who voluntarily adopted a smoke-free home, 44% still reported exposure to SHS in their homes [9]. The heightened awareness of the harms of SHS have led many MUH residents to prefer smoke-free policies in their apartments [4,7,10].

Given the harms related to SHS exposure, there was a rising momentum among federally subsidized MUH to voluntarily implement smoke-free policies. Between 1992 and 2005, 500 public housing agencies (PHA) in 27 states voluntarily implemented smoke-free policies in their properties [8]. Most of these policies were implemented in 2005 [8]. During this same time period, 64 local California jurisdictions implemented smoke-free policies in MUH [8]. Additionally, “Hawaii, Maine, Montana, New Hampshire, North Carolina, and Washington prohibited smoking in all units of all buildings financed through the states’ housing finance agencies [8]”.

In 2016, the U.S. Department of Housing and Urban Development (HUD), the federal agency that has oversight for subsidized MUH, announced a plan to implement a smoke-free policy nationwide in all HUD-owned MUH [11]. By the time HUD implemented its nation-wide smoke-free policies in PHA-owned housing, 600 PHAs had already implemented a smoke-free policy voluntarily. Once HUD’s policy went into effect in 2018, over 3300 public housing agencies (PHAs), representing 1.2 million low-income households had implemented this policy. However, the policy did not include most federally subsidized housing, including Housing Choice Vouchers, program-based vouchers, nor rentals subsidized in the private rental market, leaving a significant number of low-income residents in federally subsidized MUH at risk for SHS exposure [5]. These gaps in smoke-free policy implementation highlight a need to describe the tobacco industry’s efforts to respond to smoke-free policies in MUH in order to help policy makers in their efforts to create smoke-free living environments.

Public health scholars have described the tobacco industries’ long-standing history of fighting smoke-free policies in indoor workplaces, hospitality establishments, and other public spaces [1,2]. Scholars have shown that starting in the 1970s, the tobacco industry had used several strategies to oppose, weaken or delay implementation of smoke-free environments [1]. These strategies can be broadly categorized as: (1) direct action, such as political and media campaigns and spending on initiatives that countered tobacco control regulations [2], (2) distortion, which included funding scientific reports that contested the evidence on the harms of SHS exposure, through the now extinct Council for Tobacco Research, and Center of Indoor Air Research (CIAR) [2], (3) deflection, which included corporate responsibility efforts targeting vulnerable groups [12]; and (4) disinformation, which included advocating for accommodation policies that allowed smoking in indoor hospitality areas [13].

Previous research has shown that some of the earliest examples of direct action included the tobacco industry’s use of expensive advertisement campaigns to defeat local clean in-door ordinances. They also spent millions of dollars supporting special interest groups, organizing covert letter-writing campaigns, advocating for weaker state-wide tobacco control laws that would preempt local stronger ordinances, and lobbying congressional allies in order to block such policies [1,2]. The tobacco industry also relied on distortion, which included funding scientific reports misrepresenting the scientific evidence of SHS [14]. Starting in 1954, the tobacco industry had created their own research organizations, including the Council for Tobacco Research, Council of Tobacco Research and Center of Indoor Air Research (CIAR) [15]. These research centers supported their own peer reviewed research and included scientists who were willing to publicly speak out against the evidence of SHS harm and also support the industry in litigation [15].

The industry also used deflection, or corporate social responsibility to gain legitimacy and improve their public persona. The industry recruited and provided financial resources to social and political organizations that served populations disproportionately impacted by tobacco use such as Lesbian, Gay, Bisexual Transgender and Queer groups, African Americans social service organizations, service providers for people experiencing homelessness, and ideologically conservative libertarian groups. These groups were then deployed to protect the tobacco industry from tobacco control regulations [12]. Lastly, the industry relied on disinformation such as their push for accommodationist policies (e.g., smoking and non-smoking venues) promoted at hospitality venues. They convinced these venues based on false information that smoke-free policies would result in lost revenue and business. However, the tobacco industry ignored the harmful effects of SHS exposure among employees and clients of those venues [13].

While previous research has shown that the tobacco industry used these four primary strategies to block federal, state, and local level efforts on clean indoor air, it is unknown whether they engaged in similar strategies to respond to smoke-free policies in MUH. The primary objective of this study was to evaluate whether the tobacco industry engaged in similar tactics to respond to smoke-free policies in MUH as they had in the hospitality industry, workplaces, and public spaces. Previous studies have shown that disclosing industry tactics to targeted groups can reduce the impact of those tactics on tobacco control policies [16]. Thus, examining the tobacco industry’s efforts to respond to smoke-free policies in MUH and sharing those findings may be an important tobacco control strategy to increase support for such policies.

## 2. Methods

We searched the tobacco documents archived in the University of California San Francisco Truth Tobacco Industry Documents Library which holds over 14 million industry documents. The Truth Tobacco Industry Documents Library is a repository of previously secret documents that were released as a result of litigation between the United States and several major tobacco companies. The Truth Tobacco Industry Documents Library has 1 million documents about the industry’s advertising, manufacturing, marketing, research and political activities [1,2,12,15]. From October 2020 to January 2021, we searched the archives using a snowball search strategy, limiting the time period from 1988 to 2018. We limited the search time period from 1988 to 2018 because this was the time when MUH were implementing smoke-free policies. We used the initial search terms of “public housing”, “secondhand smoke”, “public housing authority”, “Housing and Urban Development”, “HUD”, “low-income housing”, “ETS”, “housing”, and “Center for Indoor Air Research”. Based on the initial searches, we used new search terms, including names of individuals and organizations and government entities. We narrowed our search to emails, letters, memo, and speeches, budgets and scientific reports. The search produced 3000 documents. After excluding duplicates, correspondence and other documents that included information that was either irrelevant or beyond the scope of this study, we examined 300 documents that contained our search terms. Of those 300, 30 documents emerged that provided implicit and explicit evidence related to the objectives of the study. We organized the documents by date and iteratively reviewed them to assess whether there was evidence of industry efforts to respond to the implementation of smoke-free policies in MUH. We relied on the framework used in previous documents research, focusing on direct action, distortion, deflection, and disinformation to code the documents. We coded the documents iteratively, using a code book reflecting these four industry strategies [1,2,12,13].

Of the 30 documents, 10 included information related to distortion or scientific reports funded by the industry on indoor air in housing. We categorized those scientific reports based on whether they focused on indoor smoking in the home or work and whether they were friendly or neutral to the tobacco industries’ position on SHS. To make this determination, we read the abstract of each funded project, evaluated the corresponding publication of the project, relied on other peer-reviewed characterizations of the project, and then categorized it accordingly. In instances where the scientific project tried to elevate house-hold allergens as the cause of asthma as opposed to SHS, we marked the project as friendly because it fit into the industry’s previous efforts to downplay the harms of SHS [17]. Similarly, in the instances where the project tried to identify issues such as diet as a cause of lung cancer, we marked it as friendly to the industry. In instances where the abstract and publication was unclear on whether it was neutral or friendly, we labeled it as neutral to err on the side of caution.

## 3. Results

The tobacco documents provided implicit and explicit evidence that the industry relied on two primary strategies to respond to smoke-free policies in MUH: distortion and deflection. Specifically, the tobacco industry explicitly funded research projects that downplayed the impact of SHS exposure on inner city residences (distortion) and engaged in corporate responsibility (deflection) events to partner with low-income PHAs.

### 3.1. Distortion-Funding Scientific Research

The tobacco industry was concerned about the science showing a link between SHS and asthma for low-income, inner-city residents and used CIAR to undermine this research [18]. In the 1990s, Phillip Morris relied on scientific studies to develop talking points to refute claims that SHS in the home was a danger to children [18]. They cited several scientific studies to claim that children’s respiratory symptoms could be linked to exposures such as “dampness in the home, contagious respiratory infections at home and daycare centers, heredity and even cooking with gas”, but not environmental tobacco smoke (ETS) [18].

CIAR replicated this exact strategy, funding research that downplayed the harms of ETS for inner-city housing residents [19]. In a draft 1997 document entitled, “CIAR Asthma/Allergy Workshop”, CIAR stated that “reasons to fund allergy and asthma” projects are that “asthma is increasing, especially among inner-city residents, asthma can be fatal, and some investigators are trying to show that ETS causes asthma [emphasis in original] [19]”. This same document revealed their intention to show that asthma was a “genetically determined or predisposing condition”, caused by “house dust, mites, cockroaches and triggered by cold weather, exercise, and emotions [19]”. The researchers that were featured in this 1997 draft workshop document received USD 4.2 million to fund their tobacco-related projects on indoor air quality [20]. More broadly, CIAR spent over USD 60 million on 142 non-post-doctoral research projects on indoor air quality [20]. Out of these 142 research projects, 48 (33.8%) of the studies focused on ETS directly. Of the 48 ETS projects, 14 focused on the home, 11 focused on methods and/or general air quality, 19 focused on work and home, and 4 focused exclusively on work [20]. Of the 14 ETS related research projects that focused exclusively on the impact of SHS in the home, 13 of those projects published results that were favorable to the tobacco industry’s position on SHS and one was neutral (Table 1) [20].

CIAR was disbanded in 1999 as part of the Master Settlement Agreement [26], and we did not find any documents showing that the tobacco industry continued to fund projects on air quality in housing beyond this period. In place of CIAR, the tobacco industry created other research groups such as Phillip Morris’ Life Sciences Research Office [27], and the Philip Morris External Research Program [28], and British American Tobacco’s Institute for Science and Health [28]. However, we did not find any documents after 2000 explicitly showing evidence that the tobacco industry used this tactic to respond to smoke-free policies.

### 3.2. Deflection-Corporate Responsibility

While the tobacco industry used CIAR to influence the science of indoor ETS among inner-city residence, they used the now extinct Tobacco Institute to conduct corporate responsibility activities with low-income MUH residents. Starting in 1989, the Tobacco Institute began to build these relationships with public housing residents through PHAs. The Tobacco Institute and the individual tobacco companies invested in low-income housing, and funded events in public housing that focused on youth activities and fire safety. For example, the Tobacco Institute started funding a fire safety program with The New Jersey Department of Community Affairs and Chippewa Falls Alaska fire department to install fire detectors in low-income homes [29,30]. They also targeted PHAs at the Alaska State Housing Authority and the Memphis Housing Authority to implement a fire safety program [31].

The Tobacco Institute also partnered with Memphis State University, now called Memphis University, to target public housing residents [32]. Tobacco fire consultant Tridata issued a memo to the Tobacco Institute entitled, “Assistance to Memphis State University for Public Education for Public Housing Projects”. The memo detailed the benefits of partnering with public housing, the local fire department, and the fire chief [28]. The memo stated that they hoped this program would help them make political gains with black firefighter associations, mayors of large cities and the public education community. The Tobacco Institute put the President of Tridata, Philip Schaeman, in charge of helping the professors revise their proposal and “review specific materials [33]”. The title of the program was called, “Reducing Fire Incidence in Low-Income Housing”, and they planned to have the tobacco industry representatives to meet each public housing resident as they came into the housing authority to renew their lease [28]. While there was no evidence to show that the residents met with tobacco industry personnel, the documents did show that residents over 18 years old were made aware that the tobacco industry was part of this program [33].

The tobacco industry then focused on investing in low-income housing and after-school youth programs. For example, from 1993 to 1994, Phillip Morris invested over USD 71 million in low-income housing programs and development [34,35]. RJ Reynolds also sponsored an after-school program with Winston Salem Housing Authority [36]. RJ Reynolds’ program ran parallel to the Tobacco Institute’s youth program called “Tobacco: Helping Youth Say No [37]”. The Housing Authorities of Broward County, Fayetteville County and Buffalo city requested copies of these programs based in part on advertisements the Tobacco Institute ran in Governing Magazine [38,39]. Governing magazine was a publication that provided critical information about laws, policies, and practices of state and local governments. State and local government executives and officials would read these magazines to stay informed, learn best practices, and to keep abreast of what state and local governments were doing.

In 1998, the tobacco industry considered a strategy that would transition from deflection-corporate responsibility to direct action. In a Tobacco Institute document entitled, “Indoor Air Quality in Public Housing”, the tobacco industry acknowledged that the management of public housing is run at the local level, that regulations would likely be local and that politicians would be hesitant to “invade the sanctity of the private home with smoking restrictions”. The document recommended that the tobacco industry establish relationships with “minority-rights groups”, PHAs and tenant rights groups to blame poor air quality on elected officials instead of ETS [40]. They concluded that this strategy would show that “smoking restrictions could backfire”. The Tobacco Institute did not have a chance to implement this strategy because it was disbanded in the same year that this document was produced [40]. While we did not find any documents that showed that the industry carried out these direct-action tactics once the Tobacco Institute disbanded, we did find documents that showed that the industry continued their corporate responsibility efforts targeting public housing residents.

Starting in October 2001, Phillip Morris gave USD 250,000 to the New York Knicks to target “low-income housing areas with their youth smoking prevention program [41]”. In a document entitled, “New York Knicks/Phillip Morris Healthy Lifestyle Clinics”, they detailed how they would specifically target the low-income housing projects in Harlem and the Bronx, New York [42]. The USD 250,000 investment by Phillip Morris in 2001 was one of the highest amounts they had given an organization for their youth smoking prevention programs [42]. However, the extent that Phillip Morris was involved could not be ascertained as the contract between the Knicks and Phillip Morris was marked “Restricted” under attorney-client privilege.

## 4. Discussion

The tobacco documents provided implicit and explicit evidence that the tobacco industry tried to respond to smoke-free policies in MUH using distortion and deflection. Although we did not find evidence of direct action, their distortion and deflection strategies must be viewed within their broader strategies of trying to influence smoke-free policies. The scientific projects sponsored by CIAR allowed the tobacco industry to frame SHS as less dangerous compared to other environmental triggers among low-income MUH residents. CIAR was explicit and intentional in its efforts to focus their SHS research on “inner-city residents”, a phrase that they used to describe lower-income individuals living in MUH.

CIAR focused 30% of its scientific projects on ETS in the home, relative to 3% that focused just on the workplace. Almost all the reports that focused on air quality in the home produced results favorable to the tobacco industries’ position on ETS. CIAR and the Council for Tobacco Research was disbanded in 1999, but individual tobacco companies developed their own research programs, such as Phillip Morris’ Life Sciences Research Office and the Philip Morris External Research Program, and British American Tobacco’s Institute for Science and Health [26,27,28]. While we searched the documents for connections between these newer research programs and ETS in the home, we were unable to find any that explicitly focused on MUH.

The tobacco industry also established relationships with low-income PHAs through corporate responsibility events. By partnering with low-income groups through corporate responsibility, they could establish a potential ally in fighting indoor smoking regulations [12]. They invested in low-income housing, developed youth after-school programs for low-income public housing residents and established a fire detector and safety program.

Their strategy to partner with PHAs has to be viewed within their long-held strategy of using corporate responsibility to target vulnerable groups, especially African American leadership groups [43] Previous research has found that the tobacco industry established a relationship with almost every African American civic organization “for three specific business reasons: to increase African American tobacco use, to use African Americans as a frontline force to defend industry policy positions, and to defuse tobacco control efforts [16]”. Their efforts to target minorities, which comprised 58% of public MUH, was in large part to establish this group as a frontline force against tobacco regulation, a strategy they had previously used to block other tobacco control efforts [16].

HUD, in following the model of local PHAs in implementing smoke-free policies, demonstrated that local-level initiatives could serve as a model for federal tobacco control policies to reduce SHS exposure in MUH. HUD’s policy, however, does not apply to the majority of low-income, subsidized housing, thus leaving residents in these housing units vulnerable to tobacco exposure [8]. A potential way to counter these effects are to increase local residents’ awareness of industry tactics to block such policies—a strategy that was used among African American youth [16]. This effort could spur other locally driven tobacco control efforts and serve as a model for federally directed smoke-free policies.

This study has several implications about smoke-free policies throughout the country as it relates to ETS from tobacco and other products such as vaping and cannabis. As more MUH properties become smoke-free, there will be a need to track instances where these policies may have been weakened or revoked by the tobacco, vaping, and/or cannabis industry [44,45]. Additionally, from a global health perspective, examining whether the industry similarly attempted to respond to policies around smoking in MUH in other countries could be an important future direction to consider.

This study had several limitations. We reviewed the documents that were made available and public in the tobacco documents library, and therefore may have missed documents that were not made public or were deemed privileged and confidential. However, the absence of explicit evidence of direct action or engaging in disinformation does not indicate that the industry did not engage in these strategies to influence smoke-free policies in MUH. We were limited in our ability to determine whether the tobacco industry changed, altered, or blocked smoke-free policies in MUH. Typically, it is easier to determine if the tobacco industry changed, blocked, or altered tobacco control laws when they use direct action, such as a law or initiative they defeated. Given the hyper-localized nature of MUH policies, it would be difficult to ascertain whether the tobacco industry influenced a particular apartment building’s policy.

## 5. Conclusions

Despite these limitations, we were able to describe the distortion and deflection tactics that the tobacco industry engaged in to respond MUH properties implementing smoke-free policies. However, most of these explicit efforts took place in the 1990s through early 2000s, almost 15 years after the U.S. Surgeon General made the public aware of the harms of SHS [2]. While the scientific reports were designed to downplay the harms of SHS in low-income housing, the corporate responsibility initiatives were strategic to maintain allies among a population of smokers that have a higher prevalence of smoking and lower success in quitting than the general population. Despite these industry efforts, local initiatives to implement smoke free policies in MUH were still successful and may have increased momentum for HUD to implement its smoke-free policy in public housing that they owned. Our findings suggest that similar local efforts, through advocacy from non-smokers’ rights groups, in combination with HUD’s support may have the potential to trigger widespread implementation of smoke-free policies in other types of MUH.

## Figures and Tables

**Table 1:** Center for Indoor Air and Research Funded Projects on SHS in the Home.

Title of Project	Year	Principal Investigator	Total Contract Amount
The Effects of Parental Smoking Cessation on Asthmatic Children *	1990	Samuel B. Lehrer, Ph.D. Tulane University Medical Center	USD 389,805
Charcoal Smoke and Risk of Respiratory Infections *	1991	Adolfo Correa-Villasenar, M.D., Ph.D. Johns Hopkins School of Hygiene and Public Health	USD 431,007
Development of Inhalant Allergy and Asthma in Children [21]	1994	Thomas A.E. Platts-Mills, M.D., Peter W., Heyntann, M.D. University of Virginia, Health Sciences Center	USD 590,642
A Questionnaire Study on ETS Related to Environmental Factors, Particularly Diet *	1994	Ragnar Rylander, M.D. University of Gothenburg Sweden	USD 333,031
Epidemiology and Genetics of Atopic Asthma *	1994	Morton Corn. Ph.D. Johns Hopkins University, School of Hygiene and Public Health	USD 806,892
The Relationship Between Indoor Air Quality Indicators and Asthma Manifestations in European Children with-Chronic Respiratory Symptoms [22]	1996	Bert Brunekreef, Ph.D. Wageningen Agricultural University	USD 63,586
Exposure to Biological Containments in the Residential Indoor Environment and the Development of Respiratory Allergy and Asthmatic Children *	1996	Bert Brunekreef, Ph.D. Wageningen Agricultural University	USD 314,115
Pathogenesis of Virally Induced Exacerbations of Asthma [23]	1996	David Proud, Ph.D. Johns Hopkins University, School of Medicine	USD 622,071
Pulmonary Effects of ETS Exposure on Asthmatic Subjects [24]	1997	‘Samuel B. Lehrer, Ph.D. Tulane University Medical Center	USD 1,213,630
The Effects of Parental Smoking Cessation on Asthmatic Children *	1997	‘Samuel B. Lehrer, Ph.D. Tulane University Medical Center	USD 389,805
Dust Allergens in the National Cooperative Inner-City Asthma Study *	1998	Peyton A. Eggleston, M.D. Johns Hopkins University School of Medicine	USD 174,879
Environmental Tobacco Smoke and Mortality Among CPS I [25]	1998	James E. Enstrom, Ph.D., M.P.H. University of California, Los Angeles	USD 525,000
Evaluation of Methodology for Personal Exposure Monitoring of ETS and VOCs for Korean Non-smokers *	1998	Sung-Ok Back, Ph.D. Yeungnam University, Korea	USD 150,000 (Neutral Study)
Synthesis of the Entire 16-US-City Study *	1999	Robert Tardiff, Ph.D. The Sapphire-Group, Inc. Bethesda, Maryland	USD 207,724

The table comes from CIAR document that list all of CIAR’s funded projects. Some of the projects were published in journals, others were not. According to the CIAR list, the project title was often different than the title given in the journal publication. The reference section will have the citation for the journal article associated with the project. For the projects that did not turn into publications and do not have a corresponding journal article citation, we have notated it with an * [20].

## Data Availability

All data generated or analyzed during this study are included in this published article.
